# Decreased incidence of hidradenitis suppurativa and psoriasis in diabetic patients treated with GLP-1 receptor agonists: A retrospective cohort study

**DOI:** 10.1016/j.jdin.2025.07.003

**Published:** 2025-07-24

**Authors:** Lauren M. Ching, Christopher A. Guirguis, Nicholas L. Iskandar, Joe K. Tung

**Affiliations:** a Georgetown University School of Medicine, Washington, District of Columbia; b Georgetown University Pre-College Program, Washington, District of Columbia; c University of Pittsburgh School of Medicine, Pittsburgh, Pennsylvania; d Department of Dermatology, University of Pittsburgh Medical Center, Pittsburgh, Pennsylvania

**Keywords:** diabetes, GLP-1, glucagon-like peptide-1 receptor agonists, hidradenitis suppurativa, psoriasis

GLP-1 receptor agonists (GLP-1RAs) have become increasingly prescribed for the management of type 2 diabetes mellitus (T2DM) and obesity.^1^ Beyond glycemic control and management of metabolic conditions, GLP-1RAs have shown promise in conditions such as Alzheimer dementia and obstructive sleep apnea, which are often comorbid with obesity.^2^^,^^3^ Psoriasis and hidradenitis suppurativa (HS) are inflammatory dermatologic conditions associated with obesity and its accompanying proinflammatory state.^4^ Given the antiinflammatory nature of GLP-1RAs, this study explores the effect of GLP-1RA on HS and psoriasis in patients with diabetes using a national representative database, after adjusting for effects on diabetic control and weight loss.

The All of Us Database (AoUDB) was queried for patients with T2DM, followed by diagnoses of HS or psoriasis in these patients. Prescriptions of GLP-1RA (albiglutide, dulaglutide, exenatide, liraglutide, lixisenatide, and semaglutide) and common diabetic medications (metformin, sodium-glucose cotransporter 2 inhibitors, α-glucosidase inhibitors, dipeptidyl peptidase-4 inhibitors, and sulfonylureas) were then queried. Queries included all patients enrolled from database inception (May 6, 2018) to October 31, 2023; however, retrospective data acquired from electronic health records through AoUDB have no finite beginning date. Individuals were grouped as either having any or no exposure to GLP-1RA. Multivariate regression was performed to assess the odds ratio (OR) of having HS in patients exposed to GLP-1RA, followed by a separate analysis assessing the OR of having psoriasis in patients exposed to GLP-1RA. The regression models controlled for demographic factors, socioeconomic factors, smoking history, weight, body mass index, hemoglobin A1C , diabetic complications, and prior exposure to the aforementioned diabetic medications. Individuals whose HS or psoriasis diagnosis predated their T2DM or first GLP-1RA exposure were excluded to ensure a time-dependent effect.

This study identified 74,910 patients with T2DM, of whom 1601 had psoriasis, and 601 had HS. In patients with T2DM, those treated with GLP-1RA were significantly less likely to have a future diagnosis of HS (OR = 0.61, 95% CI, 0.48-0.76, *P* < .001) (Table I). Similarly, those treated with GLP-1RA were significantly less likely to have a future diagnosis of psoriasis (OR = 0.41, 95% CI, 0.35-0.48, *P* < .001).

**Table I:** Basic demographics of analyzed cohorts

Variable	T2DM patient in HS analysis† (*n* = 74,470)	HS (*n* = 601)	T2DM patient in psoriasis analysis† (*n* = 73,225)	Psoriasis (*n* = 1601)
Race				
White, *n* (%)	34,226 (46.0)	198 (32.9)	33,268 (45.4)	999 (62.4)
Black or African American, *n* (%)	17,040 (22.9)	248 (41.3)	17,064 (23.3)	195 (12.2)
American Indian or Alaska Native, *n* (%)	1612 (2.2)	–‡	1603 (2.2)	–‡
Asian, *n* (%)	1408 (1.9)	–‡	1386 (1.9)	–‡
Other race, *n* (%)	20,184 (27.1)	142 (23.6)	19,904 (27.2)	362 (22.6)
Gender				
Male, *n* (%)	31,521 (42.3)	131 (21.8)	30,852 (42.1)	673 (42.0)
Female, *n* (%)	41,760 (56.1)	464 (77.2)	41,198 (56.3)	903 (56.4)
Other gender, *n* (%)	1189 (1.6%)	–‡	1175 (1.6)	25 (1.6)
BMI (kg/m^2^), mean (SD)	33.51 (8.23)	37.72 (9.43)	33.52 (8.25)	33.52 (8.42)
Weight (kg), mean (SD)	94.22 (24.88)	105.53 (28.16)	94.21 (24.90)	94.15 (25.11)
Age (y), mean (SD)	64.68 (13.38)	55.09 (13.09)	64.57 (13.43)	67.62 (12.59)
Age at diagnosis (y), Mean (SD)	43.75 (12.95)§	49.14 (12.84)	54.89 (12.54)§	59.78 (12.58)
Exposed to GLP-1RA				
Yes, *n* (%)	14,612 (19.6)	113 (18.8)	14,178 (19.4)	182 (11.4)
No, *n* (%)	59,858 (80.4)	488 (81.2)	59,047 (80.6)	1419 (88.6)
Unadjusted risk for developing comorbid conditionsװ				
HS, OR (95% CI)	0.95 (0.77-1.16)¶			
Psoriasis, OR (95% CI)			0.53 (0.45-0.62)∗	
Adjusted risk for developing comorbid conditions#				
HS, OR (95% CI)	0.61 (0.48-0.76)∗			
Psoriasis, OR (95% CI)			0.41 (0.35-0.48)∗	
Average time from T2DM diagnosis to comorbid condition diagnosis∗∗				
HS, median y (interquartile range)	3.98 (1.45–7.87)			
Psoriasis, median y (interquartile range)			3.44 (0.92-7.18)	

*AoUDB*, All of Us Database; *BMI*, body mass index; *GLP-1RA*, GLP-1 receptor agonists; *HS*, hidradenitis suppurativa; *OR*, odds ratio; *T2DM*, type 2 diabetes mellitus.

∗ OR significant with a *P* value < .001.

† The descriptive statistics for each T2DM cohort are different for each comorbid condition assessed (HS vs psoriasis). The exclusions are specific to the comorbid condition being assessed, meaning the final T2DM cohort after inclusions and exclusions is unique to the comorbid condition assessed. This is because the exclusion performed in each analysis encompasses any patient with the corresponding comorbid condition prior to T2DM to avoid confounding the temporal relationship.

‡ Any absolute values <20 were omitted due to privacy agreements with the AoUDB.

§ This reflects the average age of diagnosis of T2DM in the population of patients with both T2DM and the comorbid condition after excluding those with a diagnosis of the comorbid condition prior to their first diagnosis of T2DM.

װ OR Calculated using Fisher exact test with no covariates.

¶ OR had a *P* value > .05 and was therefore not significant.

# aOR calculated using multivariate regression to adjust for age, race, gender (as self-identified by participants at the time of recruitment into AoUDB), income, smoking history, weight, BMI, hemoglobin A1C, the presence of diabetic complications, adverse social determinants of health, and prior exposure to metformin, sodium-glucose cotransporter 2 inhibitors, α-glucosidase inhibitors, dipeptidyl peptidase-4 inhibitors, and sulfonylureas.

∗∗ This reported data were provided using the cohort prepared using data imputation for any missing data.

These findings support a protective effect against HS and psoriasis in patients with T2DM taking GLP-1RAs independent of weight loss, smoking status, or glycemic control. This anti-inflammatory effect may be mediated through increased anti-inflammatory cytokines (eg, interleukin [IL]-10), reduced inflammatory mediators through inhibition of NF-kB activation, and suppression of proinflammatory signals (eg, tumor necrosis factor-α, IL-6, and IL-1B) in immune cells.^5^ Limitations of this study include variance in data harmonization across sites and a lack of data indicating disease severity. Additionally, selection bias may occur in the selected population due to the exclusions to maintain a time-dependent relationship. This results in a skew toward an older cohort (Table I), as younger patients who may have both conditions would be selected against through the exclusion criteria. Future research should focus on prospective studies exploring the use of GLP-1RAs as therapeutic tools to treat HS or psoriasis, prevent disease progression, and evaluate their effect in patients without diabetes.

## Conflicts of interest

Dr Tung is or has been a clinical trials investigator, consultant, or speaker for AbbVie, Alumis, Amgen, Apogee, Arcutis, Boehringer Ingelheim, BMS, Eli Lilly, Galderma, Incyte, Johnson & Johnson, Novartis, Pfizer, Regeneron, Sanofi, Sun Pharmaceutical, Takeda, and UCB. Drs Ching, Guirguis, and Iskandar have no conflicts to declare.
